# Hot Air Treatment of Phthisis

**Published:** 1890-07-12

**Authors:** 


### THE PRACTITIONER'S MIRROR AND RETROSPECT.

*[The Editor will be glad to receive offers of co-operation and contributions from members of the profession. There is no doubt that much valuable material full of interest and instruction is at present lost to science. This is largely so in the case of (1) the younger and less-known members of the hospital staff (2) those connected with cottage hospitals, and (3) the great body of medical practitioners not attached to any institution. The co-operation of all is cordially invited to enable the Editor to make* THE HOSPITAL *of the utmost possible use and value to the profession. Specialists willing to review books on their special subjects are invited to communicate this fact at once. All letters should be addressed to* THE EDITOR, THE LODGE, PORCHESTER SQUARE, LONDON, W.]

### MEDICINE.

### HOT AIR TREATMENT OF PHTHISIS.

Some time ago we mentioned with considerable doubt the hot air treatment of phthisis, which has been advocated in certain quarters. In a recent number of the *New York Medical Record* there is an article by Dr. W. Gilman Thompson, professor of physiology in the New York University, headed "The Fallacy of the So-called Hot Air Treatment of Phthisis." The theory of the hot air treatment is based on the assumption that heat of a degree slightly above the body temperature destroys, or renders innocuous the tubercle bacilli, so that if the germs can only be heated sufficiently *in situ* by the inhalation of super-heated air, they will cease to do mischief. To accomplish this result several kinds of apparatus have been devised. Dr. Bowie reported last year in the *Lancet* that he has prescribed inhalations of air with the Weigert apparatus at 120° to 140° F. for a phthisical patient, with the effect of increasing the cough and expectoration for the first four weeks, although the general condition as to appetite, sleep, &c., improved. Several others have reported similarly. But Dr. Stern, of New York, is most enthusiastic on the subject, claiming complete cures! The Weigert apparatus seems to have been most generally used. It consists of a double copper cylinder, with a connecting tube, mouth-piece, and valves. The air within the cylinder is heated by a Bunsen burner or lamp to 482° F., but Weigert himself admits that when the air reaches the mouth-piece the temperature is only 320° F. Dr. Thompson details a number of experiments, from which he arrives at the conclusion that continuous inhalation of air heated from 200° to 300° F. at the nose does not raise the temperature of the lungs at all in some cases. In other cases, if continued for an hour or more, there may be a rise of from 2° to 4°. But this, he says, is due to various causes, other than the entrance of hot air into the alveoli. The temperature of the trachea under corresponding conditions rises only 4° to 7°. The following conditions combine to secure a uniform mean temperature of the air in the lungs, and to prevent the entrance of super-heated air: The great vascularity of the pulmonary tissue; the extensive surface of the air-sacs and blood-vessels; the slow diffusion of the tidal with the residual air; the uniform temperature of the blood; the remarkable heat-regulating mechanism of the entire body, which constantly tends to prevent local increase of temperature through the agency of a very rapid blood-stream; the low specific heat of water and of the blood, which is largely composed of water; the large quantity of blood in circulation approximately fourteen pounds per man; the enormous number of heat-units rendered latent by evaporization of water, and the ease with which water can pass from the blood plasma to the surface of the mucous membranes in considerable quantity; the conduction of heat by the body tissues. Dr. Thompson thinks that the alteration in the symptoms reported by those who have used the hot air treatment is not an argument in its favour. None of the changes have been of a decided type, and the symptoms of tuberculosis frequently vary under different conditions of diet, hygiene, and environment. Temporary improvement often occurs. And in most cases where the hot air treatment has been applied, other means have been also used. Dr. Thompson has not limited his experiments to the use of hot air, but he has also used cold air. It may, perhaps, be recollected that Tullio, of Naples, and Lipari, of Palermo, last year advocated the employment of cold air inhalations for pulmonary hæmorrhage. But, as the result of his series of experiments, Dr. Thompson asserts that cold air does not affect the temperature of the trachea or lungs, any more than hot air, and is therefore equally useless for any clinical purposes whatever. Dr. W. L. Cary, physician to the Brooklyn Throat Hospital, has also published a paper basing objections to the hot air treatment on scientific, although mainly theoretical grounds. These are chiefly that we do not find any changes in body temperature, or in the character of the respiration in those who work in great heat, nor in those exposed to arctic cold. The greatest variation in the body temperature does not exceed 5° F. Very hot air in contact with the mucous membrane would destroy the epithelium, and produce swelling and congestion. Bacilli are often buried in large tubercular masses. To reach such foci the hot air must warm the surrounding blood. Blood-serum albumin may coagulate at as low a temperature as 122° F. We fear the hot air treatment of phthisis must be consigned to that long list of remedies which have been proposed and failed in the treatment of this disease, and that we shall still be obliged to maintain our greatest confidence in pure air and other suitable environment.

